# 12-Month prevalence of stroke or chronic consequences of stroke in Germany

**DOI:** 10.17886/RKI-GBE-2017-019

**Published:** 2017-03-15

**Authors:** Markus A. Busch, Ronny Kuhnert

**Affiliations:** Robert Koch Institute, Department for Epidemiology and Health Monitoring, Berlin, Germany

**Keywords:** STROKE, DISEASE OUTCOMES, HEALTH MONITORING, GENERAL POPULATION, GERMANY

## Abstract

Stroke is the second most common cause of death globally and an important cause of disability in adults. According to the GEDA 2014/2015-EHIS study, 1.6% of adults (1.7% of women and 1.5% of men) in Germany had a stroke or chronic consequences of stroke during the past 12 months. For those aged under 55 years, the 12-month prevalence of these health problems remains well below 1% for both sexes, but then increases steeply and disproportionately to 6.3% for those aged 75 years and over. Prevalence among women with a low level of education (3.6%) is higher than among women with a high level of education (0.6%). Education has only a weak effect on prevalence among men. The indicator analysed here (12-month prevalence of stroke or chronic consequences of stroke) was developed for the European Health Interview Survey (EHIS) 2014/2015, which means that comparative data for Germany is not yet available.

## Introduction

Stroke is a disease of the brain characterised by sudden damage to brain tissue following either blockage of blood vessels (ischemic stroke) or bleeding (haemorrhagic stroke) in the brain [1, 2]. About 80-85% of strokes are ischemic strokes resulting from an acute blockage of a brain artery by a blood clot. Ischemic strokes are caused mostly by atherosclerosis of the carotid or cerebral arteries or blood clots from the heart caused by atrial fibrillation or heart valve diseases [1, 3, 4]. Haemorrhagic strokes in contrast typically occur due to ruptures of small arteries deep in the brain weakened by the long-term effects of high blood pressure. Insufficient blood supply or bleeding in the brain cause localised or generalized dysfunction to the brain, resulting in sudden neurologic symptoms. The most frequent symptoms are paresis (weakness) or paralysis and impaired sensation (numbness) of arm, leg or face (mostly limited to one side of the body), speech problems, loss of vision, dizziness, unconsciousness and severe headache.

Stroke is the second most common cause of death in Germany and globally [2, 5, 6] and one of the most frequent causes of disability in adults [2, 6, 7]. One third to one quarter of all stroke patients die within the first year after a stroke [1, 8, 9]. Up to 40% of those who survive will suffer longer-term impairments in daily activities. They may find it hard to move, wash or dress on their own or to eat [1, 2, 10-12]. In many cases, this means they will require nursing care. An analysis of claims data from statutory health insurance providers in Germany showed that the percentage of people who receive professional care increases by 13% to 19% after a stroke [13, 14].

**GEDA 2014/2015-EHIS**

**Table:** 

**Data holder:**	Robert Koch Institute
**Aims:**	to provide reliable information about the population’s health status, health-related behaviour and health care in Germany, with the possibility of a European comparison
**Method:**	questionnaires completed on paper or online
**Population:**	people aged 18 years and above with permanent residency in Germany
**Sampling:**	registry office sample; randomly selected individuals from 301 communities in Germany were invited to participate
**Participants:**	24,016 people (10,872 men; 13,144 women)
**Response rate:**	26.9%
**Study period:**	November 2014 – July 2015
**Data protection:**	all participants were informed about the study’s aims and content and about data protection, and provided their informed consent

More information is available at

www.geda-studie.de

In high-income countries, stroke mortality rates as well as the rate of new cases (incidence rate) have dropped continuously for many years [1, 2]. Demographic changes and the ageing of populations, however, have led to an increase in the absolute number of stroke patients in the population and this figure will probably continue to rise [1, 2].

Planning prevention and care will require information on the disease burden of stroke for the population. This has led to the introduction of an indicator for European health monitoring to measure the frequency and impact of stroke over time in European Union (EU) countries [15]. In the context of the European Health Interview Survey (EHIS) 2014/2015, the Robert Koch Institute’s study German Health Update (GEDA) 2014/2015 for the first time collected data on this indicator in Germany [16]. A Focus article in this edition describes European health monitoring and health indicators in more detail [17].

## Methods

GEDA 2014/2015-EHIS surveyed various diseases and health problems. ‘During the past 12 months, have you had any of the following diseases or conditions?’ the survey asked, followed by a list of diseases. Participants were also asked whether they had suffered a ‘stroke’ or ‘chronic consequences of stroke’ during the past 12 months. Participants could fill out the GEDA 2014/2015-EHIS questionnaire in writing or online.

The indicator from European health monitoring used for this analysis is defined as presence of a stroke or chronic consequences of stroke during the past twelve months [15].

The analyses are based on data from 22,599 participants aged at least 18 years with valid answers on the 12-month prevalence of stroke or chronic consequences of stroke, excluding 1,417 participants (5.9% of the entire sample) with missing data on these variables. Analyses were carried out using a weighting factor that corrected for deviations in the sample concerning gender, age, type of community and education deviations the population structure (as of 31 December 2014). The International Standard Classification of Education (ISCED) was used to make participant’s answers on education comparable [18]. A detailed description of the methodology used by GEDA 2014/2015-EHIS can be found in the article ‘German Health Update – new data for Germany and Europe’ [16] in this issue.

## Results and discussion

Overall, 1.7% of women and 1.5% of men aged over 18 years reported having had a stroke or chronic consequences of stroke during the past 12 months. The 12-month prevalence of stroke or chronic consequences of stroke is below 1% for people aged under 55 years. Prevalence then increases disproportionately to 6.4% for women and 6.1% for men in those aged 75 years and over. Women with a low level of education reported stroke or chronic consequences of stroke during the past 12 months considerably more often (3.6%) than those with high level of education (1.3%). For men, this correlation between stroke and education is far weaker.

The indicator for the 12-month prevalence of stroke or chronic consequences of stroke presented here is taken from European health monitoring and was first surveyed for Germany in GEDA 2014/2015-EHIS. The results confirm the well-known existence of a relation between stroke and older age and lower socio-economic status [2, 19]. In line with data on the lifetime prevalence of stroke, no relevant differences between genders werefound [2]. In a European comparison, variability between EU countries in the 12-month prevalence of stroke or chronic consequences of stroke was very low [17].

Due to methodological differences, prevalence as reported by the new EHIS indicator cannot be directly compared to other epidemiologic data. Firstly, the indicator for the first time combines stroke and chronic consequences of stroke. By including chronic consequences, the subjective perception of participants, which in turn is probably influenced by factors such as education and gender, has a greater weight in the mixed indicator. Secondly, unlike in most other international health surveys, the new EHIS indicators did not ask explicitly for physician-diagnosed diseases [20-22].

Instead of using the 12-month prevalence reported in the EHIS survey, epidemiologic surveys and health reporting generally report and base their comparisons on the lifetime prevalence of physician-diagnosed stroke [2, 22, 23]. For Germany, the GEDA 2009/2012 study estimated a lifetime prevalence of 2.4% for women and 2.6% for men [2]. Prevalence in Germany seems not to have changed much during the past twelve years and there are apparently no significant differences to other countries [2, 20].

Considered separately, the indicator ‘chronic consequences of stroke’ in GEDA 2014/2015-EHIS evidences a 12-month prevalence for women and men of 1.1% (data not shown). Taken on its own, this prevalence seems plausible, assuming that 40% of stroke patients suffer from chronic disabilities [1, 2] and a lifetime prevalence of 2.5% [2, 20]. As described above, the validity of information regarding self-perceived chronic consequences of stroke is difficult to evaluate.

The EHIS questionnaire resulted from a long process of consultation between the 28 EU member countries. The questions on chronic diseases, it was decided, would not ask for physician-diagnosed diseases, but for self-perceived diseases and so differed from conventional studies. For heart attack and stroke, it was additionally decided to include chronic consequences of the diseases. These decisions were based on the assumption that this would increase the survey’s relevance for public health and reduce the impact of regional differences in care supply [15-17, 24]. Furthermore, gathering data on 12-month prevalence aimed to shed a light on the current burden of these diseases and reduce recall bias in data collection [15-17, 24]. The methodological difficulties described above and lacking comparability with other sources of data, however, make it seem unlikely that EHIS indicators for stroke as well as the indicators for heart attack and coronary heart diseases (compare Fact sheet on CHD in this issue [24]) will be used in other surveys beyond EHIS.

In spite of their described limitations, the new EHIS indicators have their value for harmonised, indicator-based health monitoring in the EU that can help identify regional differences, positive and negative trends, as well as the potentials for prevention in European populations and reveal health policy fields that require further action.

## Key statements

- 1.7% of women and 1.5% of men report having had a stroke or chronic consequences of stroke during the past 12 months.
- 12-month prevalence of stroke or chronic consequences of stroke is below 1% in the age group below 55 years of age and increases disproportionately to about 6% for those aged over 75 years.
- Women with a low level of education report stroke or chronic consequences of stroke considerably more often than women with a high level of education. Prevalence among men depends far less on education.

## Figures and Tables

**Table 1:** 12-month prevalence of stroke or chronic consequences of stroke according to gender, age and level of education (n=22,599) Source: GEDA 2014/2015-EHIS

Women	%	(95%-CI)	Men	%	(95%-CI)
**Women total**	**1.7**	**(1.4-2.0)**	**Men total**	**1.5**	**(1.3-1.8)**
**Age**			**Age**		
18 – 44 years	0.3	(0.1-0.6)	18 – 44 years	0.1	(0.0-0.4)
45 – 54 years	0.5	(0.2-1.0)	45 – 54 years	0.9	(0.5-1.5)
55 – 64 years	1.3	(0.8-2.0)	55 – 64 years	1.6	(1.1-2.5)
65 – 74 years	3.4	(2.5-4.7)	65 – 74 years	3.8	(2.8-5.2)
≥75 years	6.4	(4.8-8.5)	≥75 years	6.1	(4.7-8.0)
**Education**			**Education**		
Low	**3.6**	(2.7-4.7)	Low	**1.9**	(1.3-2.6)
Medium	**1.2**	(0.9-1.6)	Medium	**1.5**	(1.2-2.0)
High	0.6	(0.4-1.1)	High	1.3	(1.0-1.7)
**Total (women and men)**	**1.6**	**(1.4-1.8)**	**Total (women and men)**	**1.6**	**(1.4-1.8)**

CI=Confidence interval
